# Chlamydia prevalence in the general population: is there a sex difference? a systematic review

**DOI:** 10.1186/1471-2334-13-534

**Published:** 2013-11-11

**Authors:** Patrick W Dielissen, Doreth AM Teunissen, Antoine LM Lagro-Janssen

**Affiliations:** 1 Department of Primary care and Community Care, Radboud University medical center, P.O. Box 9101, Nijmegen 6500HB, The Netherlands; 2 Gender and Women's Health, Radboud University medical center, Nijmegen, The Netherlands

**Keywords:** Chlamydia trachomatis, Prevalence, General population, Gender

## Abstract

**Background:**

The focus of *Chlamydia trachomatis* screening and testing lies more on women than on men. The study aim was to establish by systematic review the prevalence of urogenital *Chlamydia trachomatis* infection in men and women in the general population.

**Methods:**

Electronic databases and reference lists were searched from 2000 to 2013 using the key words “*Chlamydia trachomatis*”, “population-based study” and “disease prevalence”. Reference lists were checked. Studies were included in the analysis if *Chlamydia trachomatis* prevalence was reported for both men and women in a population-based study. Prevalence rates for men and women were described as well as highest prevalence rate by age and sex. The difference in prevalence between the sexes in each study was calculated.

**Results:**

Twenty-five studies met the inclusion criteria and quality assessment for the review. In nine of the twenty-five studies there was a statistically significant sex difference in the chlamydia prevalence. In all nine studies the prevalence of chlamydia was higher in women than in men. The prevalence for women varied from 1.1% to 10.6% and for men from 0.1% to 12.1%. The average chlamydia prevalence is highly variable between countries. The highest prevalence of chlamydia occurred predominantly in younger age groups (< 25 years). The absence of symptoms in population-based urogenital chlamydia infection is common in men and women (mean 88.5% versus 68.3%).

**Conclusions:**

The urogenital chlamydia trachomatis prevalence in the general population is more similar than dissimilar for men and women. A modest sex difference is apparent. The prevalence rates can be used to inform chlamydia screening strategies in general practice.

## Background

Urogenital *Chlamydia trachomatis* represents one of the most common bacterial sexually transmitted infections (STIs) globally. The infection is mostly asymptomatic, easily tested and single-dose treatments of oral antibiotics are readily available [1]. Early diagnosis and treatment are important to avoid transmission to partners and to prevent complications. Left untreated it can have significant and long-term complications, particularly in women. These include pelvic inflammatory disease, ectopic pregnancy, tubal factor infertility and chronic pelvic pain [2,3]. The role for this pathogen in the development of male epididymitis and orchitis is widely accepted [4]. In (chronic) prostatitis the exact role is still under debate because of the technical difficulties in localizing *Chlamydia trachomatis* to the prostate [5].

Lack of patient knowledge about chlamydia and/or the absence of symptoms of most chlamydia infections means that two important stimuli for seeking health care are absent [6-8]. Opportunistic screening is essential if control of this infection is to be achieved. Although there is an absence of evidence about the benefits of opportunistic chlamydia screening in the general population active case-finding for chlamydia is recommended [2,9,10]. General practitioners (GPs) attend to the majority of STI consultations and are the first point of contact with the health care system for most individuals in many countries and therefore ideally placed to screen patients [11,12]. Prevalence studies in the general population are important to investigate the occurrence of chlamydia infections in the population served by GPs and can be used to guide the focus of screening activities.

The research and screening is more focused on females, with the burden of disease and infertility considered to be a predominantly female problem [9,13]. For example, annual screening for all sexually active women < 25 years is recommend in the US for women and not for men [14]. In many countries, women are more the target of chlamydia screening in the general population, reducing morbidity in women and not in men [10,15-17]. Emerging evidence suggests also an effect of a chlamydia infection on male fertility although more well-designed studies are required to prove a causal factor of chlamydia infection [4]. Women are more the focus of chlamydia control activities because they have more contact with health care related to sexual health i.e. cervix smear, oral contraception, pregnancy and IUD insertion [18]. Also, women have a higher uptake rate in chlamydia screening programmes [19,20]. For example, in a register based screening programme in the Netherlands, the participation rate was significantly higher among women (21%) than among men (10.4%) [21]. Last, in the literature, for various reasons women are still more than men considered biologically and psychosocially susceptible to chlamydia infection [22,23].

Non-population-based systematic reviews have consistently shown that the pooled prevalence of genital chlamydia between males and females is similar [16,24,25]. A systematic review and meta-analysis conducted in Australia found a pooled prevalence for women < 25 years of 5% (95 CI 3.1,6.9) and a pooled prevalence for men < 30 years of 3.9% (95 CI 2.7,5.1) in community and general practice settings. Most studies included in these reviews do not compare men and women directly in a community setting or do not report the data separately [25]. The reviews also indicated important gaps of the knowledge about chlamydia prevalence particularly in men and in the general population. For example, in a systematic review of prevalence studies in the United Kingdom, only 11% of the reported prevalence estimates were from males [24]. Asymptomatic men are under-identified in these studies and they probably play an important role in sustaining the transmission of chlamydia in the population.

While *Chlamydia trachomatis* infection is an important public health issue for women, it is probably not without importance for men. The need for robust chlamydia estimates among women and men in the general population are essential to help elucidate the burden of infection. If required, GPs’ awareness of a chlamydia infection and sensitivity for active case-finding in both women and men should be increased. This systematic review examines the available literature on the prevalence of genital *Chlamydia trachomatis* infection in the general population in studies that directly compare men and women.

## Methods

### Search strategy

**Figure 1:**
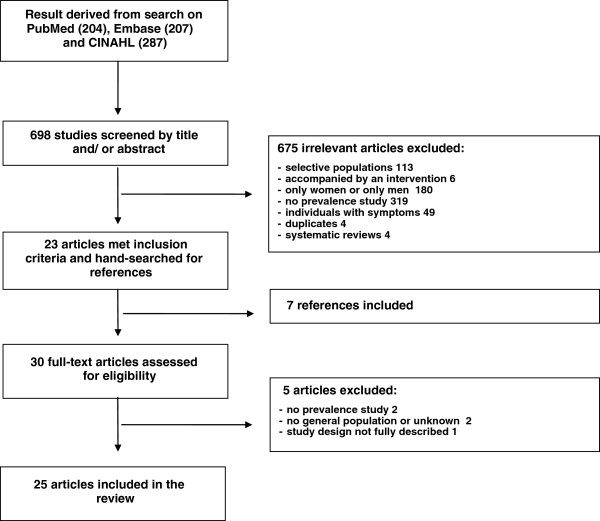
Flowchart of search, inclusions and exclusions from the systematic review.

We performed a search of the literature in the electronic bibliographic databases PubMed, Embase and CINAHL for English-language articles published between January 1, 2000 and December 31, 2012. The following search terms were used: “*Chlamydia trachomatis*” AND “prevalence” AND “population-based study OR population”. Reference lists of included articles were checked for potential studies. Also, the reference lists of selected systematic reviews were hand-searched for further publications of interest [16,24,25]. A flow chart of the search is shown in Figure 1.

### Inclusion and exclusion criteria

Studies were eligible for inclusion in the review if they (1) unambiguously reported prevalence of *Chlamydia trachomatis* infection in the general population, (2) compared and reported the chlamydia prevalence among men and women, (3) reported data from a population that was described as non-medical or non-health-care setting, (4) used nucleic acid amplification tests (NAAT) for diagnostic testing and (5) used a cross-sectional study design. We excluded articles that (1) reported only on men or women, (2) reported on selective populations, for example commercial sex workers, soldiers, ethnic groups, gynaecological patients or follow-up, (3) reported on prevalence among individuals with urogenital symptoms and (4) used serology for diagnosis. The first author (PD) applied the inclusion criteria to potentially eligible studies.

### Methodological assessment of reviewed studies

The studies were assessed and information extracted by two authors independently (PD, DT). Disagreements were resolved by discussion and consensus with the third author (TLJ). To assess the usefulness of the prevalence studies four questions were required to be answered affirmatively [26,27]: (1) is the problem being studied in the general population with data obtained from registers or data administrations independently from a health care setting?; (2) Is the study’s sampling design fully described?; (3) Was a probabilistic sample used?; (4) Is prevalence broken down by sex? Further assessment of the quality of the studies included the response rate of the total sample and sex subgroups (major flaw if < 40%), reporting number screened, reporting of prevalence type and total prevalence, prevalence in subgroups and precision of estimation (95% CI, error) [26]. If necessary, authors were contacted by email for additional information for example the origin of the registries or administration database [28,29].

The prevalence for both men and women, and if available the highest prevalence rate by age and sex, were extracted. We calculated the difference in prevalence between the sexes for each study and its 95% confidence intervals [30]. The data were not pooled for meta-analysis. It was likely that there would be considerable heterogeneity between studies making any formal meta-analysis less valid. Also, it was not considered important to the interpretation of the data because of the different geographical location of each study and therefore of limited clinical added value of this pooled prevalence rate to GPs.

## Results

### Description of included studies

Our literature searches identified 698 studies which were screened by title and abstract. The full text of thirty-seven articles was read and fourteen were excluded. Of the remaining twenty-three articles the reference lists were searched and we included seven additional studies. Thirty studies were assessed for eligibility. Twenty-five articles were included in the review. (Figure 1 and Table 1).

**Table 1:** Characteristics population-based studies reporting prevalence of Chlamydia trachomatis for both men and women

**Authors (publication year) [Reference]**	**Country**	**Men tested**	**Women tested**	**Response rate**	**Sampling**	**Test M**	**Test F**	**Invitation**	**Geographic area**
**Valkengoed (2000)**[31]	Netherlands	1908	2902	M 33.0% F 51.0%	General practices	PCR urine	PCR urine	By mail	Urban
**Fenton (2001)**[32]	UK	1474	2055	Total 71.0%	General community	LCR urine	LCR urine	By mail	General population
**Obasi (2001)**[33]	Tanzania	4749	4686	Total 80%	General community	PCR urine	PCR urine	By interviewer	Rural
**Turner(2002)**[34]	USA	244	335	Total 79.5%	General community	LCR urine	LCR urine	By interviewer	Urban
**Miller (2004)**[35]	USA	6767	7555	Total 87.6%	Schools	LCR urine	LCR urine	By interviewer	Urban and rural
**Joyee (2004)**[36]	India	603	841	NS	General community	PCR urine	PCR urine	By interviewer	Urban and rural
**Latif (2004)**[37]	Australia	525	694	M 43.1% F 56.9%	General community	PCR FVU	PCR VVS	By interviewer	Rural
**Klavs (2004)**[29]	Slovenia	683	764	M 50.9% F 60.0%	General community	PCR urine	PCR urine	By interviewer	General population
**Bergen (2005)**[38]	Netherlands	2930	5453	M 33.0% F 47.0%	General community	PCR urine	PCR urine	By mail	Urban and rural
**Götz (2005)**[39]	Netherlands	1999	4304	Total 41.0%	General community	PCR urine	PCR urine	By mail	Urban and rural
**Macleod (2005)**[40]	UK	1930	2801	M 26.6% F 36.4%	General practices	PCR urine	PCR urine VVS	By mail	Urban and rural
**Low (2007)**[19]	UK	1396	1869	M 29.5% F 39.5%	General practices	PCR urine	PCR VVS	By mail	Urban
**Deblina Datta (2007)**[41]	USA	3096	3536	Total 83.0%	General community	LCR urine	LCR urine	By interviewer	General population
**Stein (2008)**[42]	USA	5074	5854	Total 88.6%	Schools	LCR urine	LCR urine	By interviewer	NS
**Adams (2008)**[43]	Barbados	190	207	M 79.0% F 86.0%	General community	PCR urine	PCR urine	By interviewer	NS
**Uusküla (2008)**[44]	Estonia	215	345	M 32.0% F 48.0%	General community	PCR FVU	PCR VVS	By mail	Urban and rural
**Beydoun (2010)**[45]	USA	2447	3164	NS	General community	NAAT urine	NAAT urine	By interviewer	NS
**Imai (2010)**[46]	Japan	2595	4003	Total 81.5%	Schools	PCR urine	PCR urine	By interviewer	NS
**Goulet (2010)**[47]	France	1135	1445	M 65.0% F 71.0%	General community	PCR urine	PCR VVS	By interviewer	Urban and rural
**Parish (2011)**[48]	China	1138	1235	Total 69.0%	General community	LCR urine	LCR urine	By interviewer	Urban and rural
**Desai (2011)**[28]	Germany	952	855	NS	General community	NAAT urine	NAAT urine	NS	General population
**Bozicevic (2011)**[49]	Croatia	123	151	M 27.9% F 37.5%	General community	PCR urine	PCR urine	By interviewer	Urban and rural
**Eggleston (2011)**[50]	USA	798	1322	M 17.7% F 26.5%	General community	NAAT urine	NAAT urine	By interviewer	Urban
**Gravningen (2012)**[51]	Norway	466	565	M 28.8% F 34.9%	Schools	PCR FVU	PCR FVU	Class-wise	Rural
**Klovstad (2012)**[52]	Norway	605	930	M 11.9% F 18.9%	General community	NAAT FVU	NAAT FVU	By mail	Urban and rural

NS: data not stated.

PCR: polymerase chain reaction.

LCR: ligase chain reaction.

FVU: first voided urine.

VVS: vulvovaginal swab.

NAAT: nucleic acid amplification technique for example, DBProbeTec amplified DNA assay and APTIMA Combo2 assay.

The majority of the included studies were conducted in the general community (18), three were conducted in general practice [19,31,40] and four in schools [35,42,46,51]. We included the studies that recruited participants from general practices in the Netherlands and the UK because in these countries the vast majority of its population is registered at one general practice [53]. We included studies from Europe (13) [19,28,29,31,32,38-40,44,47],[49,51,52], the United States of America (6) [34,35,41,42,45,50], Asia [36,46,48], Africa [33], Australia [37] and the Caribbean [43].

Study sample sizes were moderate. The number of individuals tested in each study varied considerably, ranging between 123 and 7555 participants. In all studies but two the number of female participants was higher than male participants [28,33]. Most studies reported 95% confidence intervals for their prevalence rates of chlamydia infection in men and women but only ten studies conducted adequately statistically comparison of prevalence rate by sex [28,33,35,36,38,39,43,45],[49,50]. Also, the age groups chosen varied considerably, including individuals at puberty or adolescence to adulthood (minimum 12 years and maximum 67 years). The response rates varied in men from 11.9% to 79.0% and in women from 18.9% to 86.0%. Where reported, response rate in all studies was higher for women than for men. Only eleven studies reported total response (31.5% to 88.6%) [32-35,39,41,42,46,48,50,51]. In three studies the total response rate or response rate for men and women separately was below 40% (major flaw) [19,40,49]. In four studies the response rate for men was below 40% only [31,38,44,52]. Of the seven studies reporting low participation rates, six studies invited participants by mail [19,31,38,40,44,49,52].

The most commonly utilized sample was urine or first voided urine for both men and women. First voided urine samples were tested for all of the male population. For women, in five studies a self-obtained lower vaginal swab was used or offered [19,29,40,44,47]. The prevalence of symptoms was reported in only eight studies [19,33,35,38,42-44,52].

### Prevalence of chlamydia infection by sex

The reported prevalence of chlamydia infection among men and women in population-based settings in various countries was diverse, ranging from 0.1% to 12.1% in men and from 1.1% to 10.6% in women. Table 2 shows the prevalence of the included studies by sex and the calculated difference in prevalence between the sexes of each study. We found a statistically significant difference in chlamydia prevalence in nine studies [28,33-35,38,42,44,46,51]. The study characteristics of these nine studies did not differ from the other studies in terms of geographic area, number of participants tested, age or response rate. Black women and men were disproportionately affected. Three studies reported particularly high chlamydia prevalence in black participants compared to nonblack participants with rates generally higher in women than in men [34,35,38]. For example, Miller et al. found a chlamydia prevalence in black men of 11.12% and in black women of 13.95% compared to a prevalence in white men of 1.38% and in white women of 2.52% [35].

**Table 2:** Results population-based studies reporting prevalence of Chlamydia trachomatis for both men and women

**Authors (publication year) [Reference]**	**Overall**	**Men**	**Women**	**Prevalence difference by sex (p or OR)**	**Calculated risk difference by sex (M-F)**	**95% CI**
	**% (95% CI)**	**% (95% CI)**	**% (95% CI)**			
**Valkengoed (2000)**[31]	NS	2.4 (1.7-3.0)	2.8 (2.2-3.4)	NS	−0.4	−1.31 ; 0.51
**Fenton (2001)**[32]	NS	2.2 (1.5-3.2)	1.5 (1.1-2.1)	NS	0.7	−0.21 ; 1.62
**Obasi (2001)**[33]	1.8 (NS)	1.0 (0.8-1.4)	2.4 (2.3-2.9)	OR 2.4 (NS)	−1.4	−1.92 ; -0.88†
**Turner(2002)**[34]	3.0 (SE 0.8)	1.6 (NS)	4.3 (NS)	NS	−2.7	−5.38 ; -0.02†
**Miller (2004)**[35]	4.2 (3.5-4.9)	3.7 (2.9-4.6)	4.7 (3.9-5.7)	OR 1.3(1.0-1.6)	−1.0	−1.66 ; -0.34†
**Joyee (2004)**[36]	1.1 (0.5-1.7)	1.2 (0.4-2.0)	1.1 (0.5-1.7)	P > 0.05	0.1	−1.02 ; 1.22
**Latif (2004)**[37]	9.6 (NS)	9.0 (NS)	10.0 (NS)	NS	−1.0	−4.02 ; 2.02
**Klavs (2004)**[29]	NS	3.0 (1.9-4.6)	1.6 (1.0-2.7)	NS	1.4	−0.33 ; 3.13
**Bergen (2005)**[38]	2.0 (1.7-2.3)	1.5 (1.1-1.9)	2.5 (2.0-3.0)	P < 0.001	−1.0	−1.60 ; -0.39†
**Götz (2005)**[39]	2.4 (2.1-2.8)	2.0 (1.4-2.7)	2.6 (2.2-3.2)	OR 1.4; P = 0.08	−0.6	−1.38 ; 0.18
**Macleod (2005)**[40]	3.0 (2.3-3.9)	2.8 (2.2-3.4)	3.6 (3.1-4.9)	NS	−0.8	−1.81 ; 0.21
**Low (2007)**[19]	NS	5.3 (4.4-6.3)	6.2 (4.9-7.8)	NS	−0.9	−2.51 ; 0.71
**Deblina Datta (2007)**[41]	2.2 (1.8-2.8)	2.0 (1.6-2.5)	2.5 (1.8-3.4)	NS	−0.5	−1.21 ; 0.21
**Stein (2008)**[42]	NS	3.9 (3.1-4.8)	5.1 (4.2-6.0)	NS	−1.2	−1.98 ; -0.43†
**Adams (2008)**[43]	11.3 (8.4-14.2)	12.1 (7.7-16.5)	10.6 (6.7-14.5)	P = 0.643	1.5	−4.75 ; 7.75
**Uusküla (2008)**[44]	5.4 (3.0-7.5)*	2.7 (0.3-5.0)*	6.9 (3.6-10.3)*	NS	−4.2	−7.64 ; -0.76†
**Beydoun (2010)**[45]	1.6 (1.3-1.9)	1.7 (NS)	1.6 (NS)	P = 0.8	0.1	−0.57 ; 0.77
**Imai (2010)**[46]	NS	6.7 (NS)	9.5 (NS)	NS	−2.8	−4.12 ; -1.48†
**Goulet (2010)**[47]	NS	1.4 (0.8-2.6)	1.6 (1.0-2.5)	NS	−0.2	−1.14 ; 0.74
**Parish (2011)**[48]	NS	2.1 (1.3-3.3)*	2.6 (1.6-4.1)*	NS	−0.5	−1.72 ; 0.72
**Desai (2011)**[28]	0.9 (0.5-1.3)	0.1 (0.0-0.3)	1.8 (0.9-2.6)	P < 0.001	−1.7	−2.61 ; -0.79†
**Bozicevic (2011)**[49]	6.2 (3.3-9.1)	7.3 (NS)	5.3 (NS)	P = 0.491	2.0	−3.82 ; 7.82
**Eggleston (2011)**[50]	3.9 (2.8-5.0)	4.5 (2.4-6.5)	3.4 (2.2-4.6)	OR 0.6; P = 0.16	1.1	−0.64 ; 2.84
**Gravningen (2012)**[51]	4.1 (3.3-5.3)	3.9 (2.3-6.0)	7.3 (5.3-9.7)	NS	−3.4	−6.17 ; -0.63†
**Klovstad (2012)**[52]	5.5 (4.5-6.8)	5.1 (3.8-6.8)	5.8 (4.5-6.8)	NS	−0.7	−3.01 ; 1.61

*: Prevalences after weighting for the population distribution.

†: statistically significant difference.

NS: Data not stated.

CI: 95% confidence interval.

SE: Standard error.

In urban settings, chlamydia prevalence for women ranged from 2.8-6.2% and for men from 1.6-5.3% (Table 2). In rural settings, chlamydia prevalence for women ranged from 1.6-7.3% and for men from 1.0-3.9%. Prevalence rate for women was highest in a study with participants aged 15–20 years. Ten studies reported on chlamydia prevalence estimates from studies conducted in rural and urban settings. Chlamydia prevalence for women ranged from 1.1-6.9% and for men from 1.2-7.3%. Highest prevalence rates in women and men were in studies with predominantly adolescents and small sample sizes.

The five studies that used self-obtained vaginal swabs in women and first voided urine in men, found no statistically significant difference in chlamydia prevalence in men and women (Table 2) [19,37,40,44,47]. Reported prevalence estimates were 1.6–6.9% for women and 1.4–5.3% for men. In sixteen studies first voided urine was used in both women and men as test specimen. In these studies, chlamydia prevalence in women ranged from 1.1-10.6% and in men from 0.1-12.1%.

### Prevalence of chlamydia infection by sex and age group

Age-specific rates of chlamydia prevalence were reported in fourteen studies (Table 3) [29,31-33,36,38,40-42,45,47,48],[51,52]. Unfortunately, the reporting of chlamydia prevalence among the different age groups was diverse and the studies used variable age groups making comparison between studies difficult. For example, not all studies restricted age groups to cohorts of five years.

**Table 3:** Results population-based studies reporting prevalence of Chlamydia trachomatis for sex related to age

**Authors (publication year) [Reference]**	**Age group**	**Highest prevalence by age in men**	**Prevalence % (95% CI)**	**Highest prevalence by age in women**	**Prevalence % (95% CI)**	**sex difference by age**
**Valkengoed (2000)**[31]	15-40	21-25	3.3 (1.0-5.5)	21-25	4.4 (2.6-6.3)	↔
**Fenton (2001)**[32]	18-44	25-34	3.0 (1.7-5.1)	18-24	3.0 (1.7-5.0)	↑
**Obasi (2001)**[33]	15-19	18	1.8 (1.0-2.8)	19	3.2 (2.2-4.5)	↓
**Turner(2002)**[34]	18-35	18-20*	8.0 (SE 3.9)	18-20*	8.0 (SE 3.9)	-
**Miller (2004)**[35]	18-26	20-21*	4.7 (3.6-6.2)	20-21*	4.7 (3.6-6.2)	-
**Joyee (2004)**[36]	15-45	31-45	2.1 (0.0-5.0)	31-35	1.9 (0.0-3.9)	↑
**Latif (2004)**[37]	18-49	ns		ns		-
**Klavs (2004)**[29]	13-67	18-24	4.1 (2.2-7.4)	18-24	4.1 (2.2-7.4)	↔
**Bergen (2005)**[38]	15-29	25-29	4.1 (2.1-6.2)	15-19	4.3 (1.5-7.0)	↑
**Götz (2005)**[39]	15-29	15-19*	3.1	15-19*	3.1	-
**Macleod (2005)**[40]	16-39	20-24	5.3 (4.4-6.3)	20-24	6.2 (4.9-7.8)	↔
**Low (2007)**[19]	16-24	ns		ns		-
**Deblina Datta (2007)**[41]	14-39	20-29	3.2 (2.4-4.3)	14-19	4.6 (3.7-5.8)	↑
**Stein (2008)**[42]	18-26	18-24	1.0 (0.6-1.5)	25-26	2.1 (1.3-3.5)	↓
**Adams (2008)**[43]	18-35	18-20*	19.8	18-20*	19.8	-
**Uusküla (2008)**[44]	18-35	ns		ns		-
**Beydoun (2010)**[45]	14-39	<25	2.7 (SE 0.6)	<25	2.8 (SE 0.7)	↔
**Imai (2010)**[46]	18-39	20	8.3	19	12.2	-
**Goulet (2010)**[47]	18-44	25-29	2.7 (0.8-8.0)	18-24	3.6 (1.9-6.8)	↑
**Parish (2011)**[48]	20-64	25-34	3.9 (1.8-8.2)	35-44	4.2 (2.7-6.7)	↓
**Desai (2011)**[28]	12-17	ns		17	3.7	-
**Bozicevic (2011)**[49]	18-25	ns		ns		-
**Eggleston (2011)**[50]	15-35	15-19*	8.0 (4.3-11.6)	15-19*	8.0 (4.3-11.6)	-
**Gravningen (2012)**[51]	15-20	19-20	7.1	19-20	11.1	↔
**Klovstad (2012)**[52]	18-25	18-21	6.3 (3.9-10.0)	18-21	6.6 (4.7-9.3)	↔

*: Reported by age only, not age and sex.

ns: Data not stated.

↔: No sex difference in Chlamydia prevalence related to age.

↑: Chlamydia prevalence related to age highest in men.

↓: Chlamydia prevalence related to age highest in women.

Overall, in both men and women young age groups (<25 years) have higher chlamydia prevalence rates than older age groups but chlamydia prevalence in young age groups is not different for men and women. In six studies the highest prevalence of chlamydia infection was in the same age group for men and women [29,31,40,45,51,52]. In five studies women compared to men had the highest chlamydia prevalence at a younger age group [32,36,38,41,47].

Furthermore, in all fourteen studies but two, the highest chlamydia prevalence occurred among participants < 25 years of age; in women ranging from 2.1-6.6% and in men from 1.0-6.3%. In two studies the highest age group was > 25 years in both men and women. One study, originated from India, reported the highest chlamydia prevalence in men 31–45 years of age and in women 31–35 years of age (2.1% versus 1.9%). In a study from China, the highest chlamydia prevalence was found in men aged 25–34 years and in women aged 35–44 years (3.9% versus 4.2%) [36,48].

### The presence of symptoms by sex

There were eight studies that reported information on the presence of symptoms among individuals tested [19,33,35,38,42-44,52]. The absence of symptoms in urogenital chlamydia infections varied in men from 73.9% to 94.6% and in women from 45.3% to 95.5%. In all studies but one [35], the absence of symptoms was higher in men than in women.

## Discussion

### Statement of the principal findings

In our systematic review of 25 studies, we found modest sex differences in the prevalence of chlamydia infection between men and women in the general population. The prevalence for women varied from 1.1% to 10.6% and for men from 0.1% to 12.1%. Nine out of twenty-five studies showed a statistically significant higher prevalence of chlamydia among women compared to men. The majority of the included studies did not found a sex difference. Where studies reported age-based estimates, younger participants had higher prevalence than older participants but this is the case for both men and women. Also, the prevalence of *Chlamydia trachomatis* in urban and rural areas did not differ between men and women. The review highlighted that chlamydia prevalence was highly variable between countries but with rates often as high in men as in women. The commonly held assumption that chlamydia infection is more prevalent in women than in men should therefore be reconsidered.

### Strengths and weaknesses of the study

Two methodological issues need to be addressed as they may influenced the validity of these findings. Firstly, the majority of the studies had low or unspecified participation rates more for men than for women (< 60%). A low participation rate can induce non-participation bias and the population sample under study may not be generalizable to the whole population [54]. Few studies collected data on non-respondents for comparison with respondernts to assess the implications of study nonparticipation. It is uncertain how and if non-participation bias influenced the results. Secondly, most studies used first voided urine in women instead of vulvovaginal swabs. Both specimens from women are suitable but the vulvovaginal swab is the specimen of choice in women [55]. The number of infections identified in women in the studies using first voided urine may have been higher when using a vulvovaginal swab. The sensitivity of first voided urine for the detection of chlamydia infection in women is lower than vaginal specimens. Last we did not address specific ethnic backgrounds in our review. Few studies presented chlamydia infection by ethnical background. Ethnicity is a known risk factor for STIs [34,35].

The strengths of this review are that, in our opinion, it provides the most comprehensive review to date of chlamydia prevalence estimates in the general population directly comparing men and women. We conducted comprehensive literature searches of multiple databases and used rigorous methods to appraise the articles. It is unlikely that we excluded important articles in this field. All studies used the nucleic acid amplification test with high sensitivity and specificity not only greatly enhancing the acceptability of a screening intervention but also increasing the reliability of the prevalence estimates. It is the first review in this area to confirm the previous conclusions in other systematic reviews of chlamydia prevalence estimates in non-population-based studies that the prevalence of urogenital chlamydia infections is as high in men as in women [16,24,25].

### Implications

It is clear from this review that men are also an important reservoir of chlamydia infection for women and as such men should also be targeted for chlamydial screening. The vulnerability of women for a urogenital chlamydia infection in different countries did not emerge as a robust trend in this review of the literature. The reason for the reported sex differences are explained in the literature as artefacts or bias of reporting and the fact that women are more studied and more tested than men [16,24,25]. Chlamydia screening is frequently performed in women even in the absence of symptoms for example in routine gynaecologic care, Pap test, IUD insertion or unintended pregnancy, and it is more common to perform population-based chlamydia screening activities for women [13,14]. By contrast, fewer men are diagnosed in primary care: men are often treated for chlamydia without a definitive diagnosis, based upon symptoms such as urethritis or being a contact of a women with a chlamydia infection [56,57]. Also this review shows that men consistently have lower participation rates in chlamydia prevalence studies. The results of this study confirm and strongly support the need for higher coverage of men in chlamydia screening and research activities [4,24].

The conclusion from this review is that the prevalence of chlamydia infection in men and women in the general population is more similar rather than dissimilar regardless of age or level of urbanisation. According to the literature, many factors shape women’s and men’s risks for chlamydia infection differently [22,23,58]. Biological predisposition and certain gender-specific behaviours are mentioned in the literature as possible reasons for women being more at risk for chlamydia and other STIs than men [58,59]. For example, cervical ectopy, especially in young women, may increase their susceptibility to chlamydia infection. Cervical ectopy is more common in women using oral contraceptives [60]. Hormonal contraceptives are associated with an increased risk of chlamydia infection [60-62]. Vaginal douching is associated with bacterial vaginosis and HIV, both increasing the risk for other STIs [63]. A direct association between douching and chlamydia is less consistent [64]. In the male however, little is known about analogous phsysiological changes that might affect a man’s risk of infection with chlamydia. Circumcision in men, not in women, appears to reduce the risk of acquiring STI [65]. Men tend disproportionately to place monogamous women at risk for STI [58]. All this may be true, in the general population it seems to result in a modest difference in the prevalence of chlamydia among men and women only.

## Conclusion

We found modest sex differences in prevalence rates amongst general populations but the prevalence of chlamydia infection in men and women is more similar than they are dissimilar. The prevalence of *Chlamydia trachomatis* in population-based studies ranges from 0.1% to 12.1% in men and from 1.1% to 10.6% in women depending upon age and country. The prevalence rates can be used to inform chlamydia screening strategies in general practice in men and women given that the most serious long term consequence of chlamydia for individual women, infertility, is one that will ultimately affect also men.

## Competing interests

The author(s) declare that they have no competing interests. The authors alone are responsible for the content and writing of the paper.

## Authors’ contribution

PD was responsible for the data collection, data analysis, interpretation and writing the paper. DT contributed to the data collection and data analysis. All authors were responsible for the study design, interpretation and commenting on drafts of the paper. All authors have read and approved the final manuscript.

## Pre-publication history

The pre-publication history for this paper can be accessed here:

http://www.biomedcentral.com/1471-2334/13/534/prepub
